# An Improved Calibration Method for a Rotating 2D LIDAR System

**DOI:** 10.3390/s18020497

**Published:** 2018-02-07

**Authors:** Yadan Zeng, Heng Yu, Houde Dai, Shuang Song, Mingqiang Lin, Bo Sun, Wei Jiang, Max Q.-H. Meng

**Affiliations:** 1 Quanzhou Institute of Equipment Manufacturing, Haixi Institutes, Chinese Academy of Sciences, Jinjiang 362200, China; zyadan@fjirsm.ac.cn (Y.Z.); yhxiaoyanjing@foxmail.com (H.Y.); kdlmq@fjirsm.ac.cn (M.L.); chmh78963@163.com (W.J.); 2 College of Electrical and Control Engineering, North University of China, Taiyuan 030051, China; 3 Harbin Institute of Technology Shenzhen Graduate School, Shenzhen 518000, China; max@ee.cuhk.edu.hk; 4 Suzhou Sino-Germany Robooster Intelligent Technology Co., Ltd., Suzhou 215000, China; bo.sun@roboostertech.com; 5 Department of Electric Engineering, Chinese University of Hong Kong, Hong Kong, China

**Keywords:** light detection and ranging (LIDAR), rotating unit, Pan-Tilt Unit (PTU), bias angle, calibration, LM algorithm

## Abstract

This paper presents an improved calibration method of a rotating two-dimensional light detection and ranging (R2D-LIDAR) system, which can obtain the 3D scanning map of the surroundings. The proposed R2D-LIDAR system, composed of a 2D LIDAR and a rotating unit, is pervasively used in the field of robotics owing to its low cost and dense scanning data. Nevertheless, the R2D-LIDAR system must be calibrated before building the geometric model because there are assembled deviation and abrasion between the 2D LIDAR and the rotating unit. Hence, the calibration procedures should contain both the adjustment between the two devices and the bias of 2D LIDAR itself. The main purpose of this work is to resolve the 2D LIDAR bias issue with a flat plane based on the Levenberg–Marquardt (LM) algorithm. Experimental results for the calibration of the R2D-LIDAR system prove the reliability of this strategy to accurately estimate sensor offsets with the error range from −15 mm to 15 mm for the performance of capturing scans.

## 1. Introduction

Light detection and ranging (LIDAR) is a substantial contributing sensor for autonomous driving [1,2], 3D reconstruction [3,4,5], simultaneous localization and mapping (SLAM) [6,7,8,9,10] and visual navigation [11,12,13,14], etc. With the benefits of high accuracy and ignoring of background illumination, the rotating two-dimensional light detection and ranging (R2D-LIDAR) becomes an attractive sensor for outdoor mobile robots [15]. While some 3D LIDAR sensors, such as HDLE-32E (Velodyne Inc., San Jose, CA, USA) [16] and RS-LIDAR (Robosense Inc., Shenzhen, China) [17], have been widely adopted to capture the 3D scan of surroundings. Meanwhile, a specially R2D-LIDAR system, which was composed of a 2D LIDAR and a rotating unit, performs more dense point cloud data with a low-cost prototype. Thus, such a R2D-LIDAR system is a good choice for most applications.

Nevertheless, the skewing of the two devices, i.e., the 2D LIDAR and rotating unit, is a critical problem in the R2D-LIDAR system. To solve the skewing with two center excursion problems, Alismail et al. [15] adopted the iterative closest point (ICP) algorithm to discover the transformation matrix of the two devices’ coordinate systems. Using two cost functions through bidirectional rotations out of a simple plane; Jaehyeon et al. [18] made the calculation easier by decoupling the 6-DOF (degrees of freedom) transformations of the two devices’ coordinate systems into a 3-DOF translation and a 3-DOF rotation. Furthermore, the calibration strategy of Sosukeet et al. [19] was applied by calculating the redundancy measurement error. These works were focused on the skewing calibration between the 2D LIDAR and the rotating unit, whereas ignoring the bias of the devices themselves.

Petr et al. [20] found that the assembling and lack of calibration is the causes of the 2D LIDAR shifted problem. Hence, the system was calibrated combining with a camera to acquire the coordinate transformation. Gong et al. [21] adopted the isomorphism constraint among the laser scanner data to optimize the calibration parameters, and then used the ambiguity judgment algorithm to solve the mismatch problem. Since the work [15] performs accurate and convenient while neglecting the bias of 2D LIDAR, our approach is derived from the work [15] with the additional adjustment of LIDAR bias. In this way, the total calibration just needs to capture the point cloud data by rotating the device a full circle at one time. Subsequently, after compensating the bias of LIDAR, the method in the work [15] was applied to receive the transformation between the 2D LIDAR and rotating unit. Moreover, the bias calibration of our strategy required a flat plane to seek the bias of the 2D LIDAR centerline based on the Levenberg–Marquardt (LM) algorithm [22].

The remainder of this paper is structured as follows. Section 2 presents the working principle of the R2D-LIDAR system. The calibration procedures with LM optimizing are described in-depth in Section 3, which is then followed by the experimental validation of our proposed algorithm in Section 4. Finally, the conclusions of the work are outlined in Section 5.

## 2. R2D-LIDAR system

**Figure 1:**
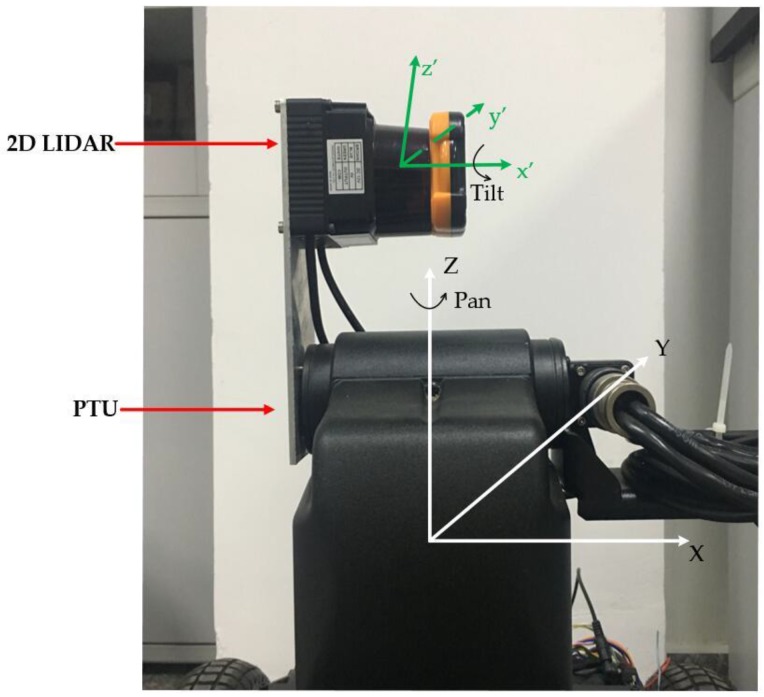
The rotating two-dimensional light detection and ranging (R2D-LIDAR) system composed by a 2D LIDAR and a Pan-Tilt Unit (PTU).

**Figure 2:**
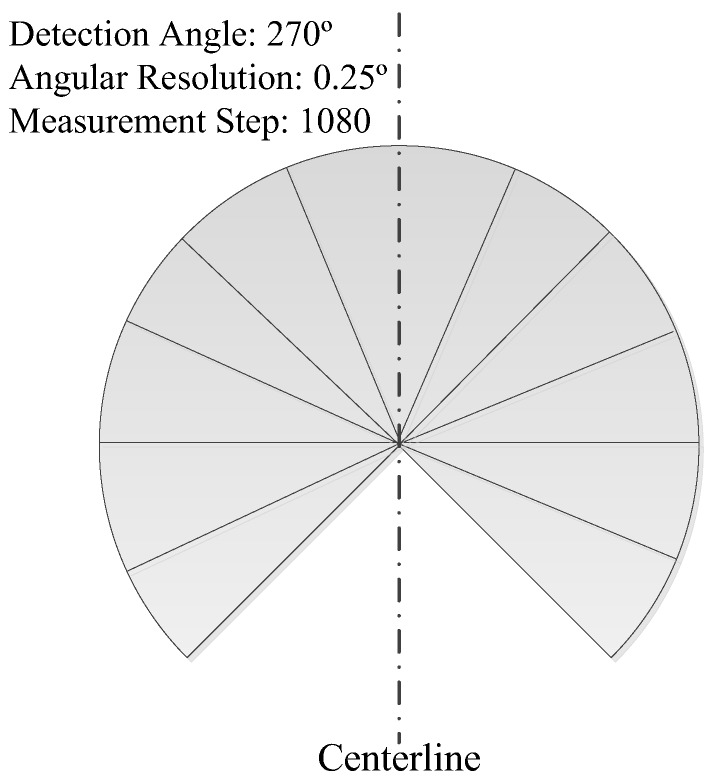
Detection range of the 2D LIDAR expresses a sector spanning 270° with 0.25° angular resolution. The detecting plane of the LIDAR that is assembled vertically result from such structure.

As shown in Figure 1, the R2D-LIDAR system employed in this paper consists of a 2D LIDAR and a Pan-Tilt Unit (PTU). The PTU yaws the 2D LIDAR to produce a proper R2D-LIDAR. The 2D LIDAR UTM-30LX (Hokuyo Inc., Osaka, Japan) used in our system that can scan 270° in a plane with the angular resolution of 0.25°. The centerline was employed to indicate the vertical direction where the scan can be divided into two parts, as illustrated in Figure 2. The PTU is PTU-D48E (FLIR Inc., Wilsonville, OR, USA) with 360°-continuous Pan and 120°-continuous Tilt range. The resolution can be determined according to the system requirement without greater than a 0.006° limit. The Pan and Tilt are used to rotate the 2D LIDAR horizontally and adjust the bias of the 2D LIDAR, respectively.

**Figure 3:**
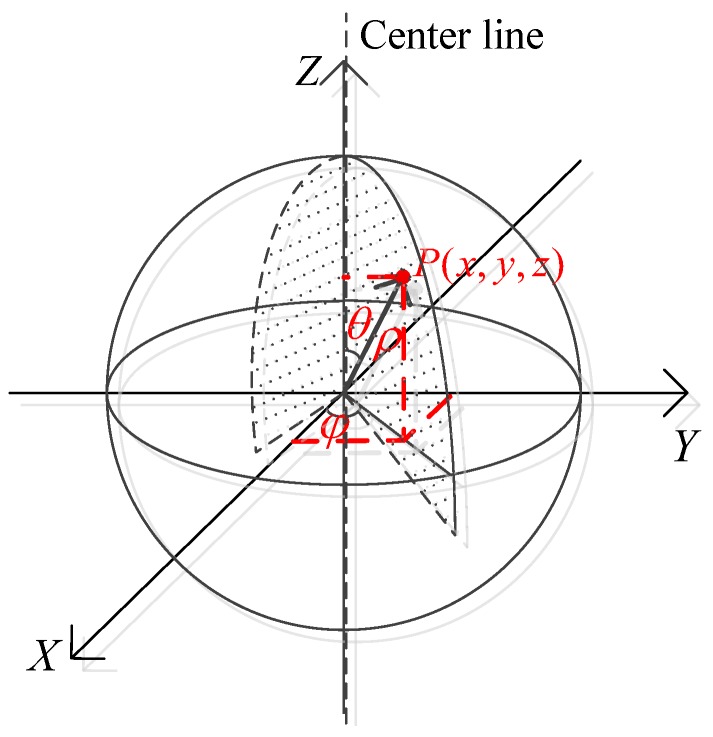
Coordinate system in the ideal situation.

To fully and more quickly capture the 3D point cloud of a space object, the work plane of the 2D LIDAR is placed vertically with the pan of the PTU. The 2D LIDAR is vertical which makes the PTU only need to rotate 180º to achieve 3D scan as shown in Figure 1. The spherical coordinate system is used to represent the position of the point cloud as illustrated in Figure 3. *ρ* is the distance measured by the 2D LIDAR, *θ* is derived via the step angle within a scan of the 2D LIDAR and *ϕ* is the rotation angle of PTU. Therefore, the Cartesian rectangular coordinates of the point cloud could be expressed as follows:

(1) $$\left\{ \begin{array}{l} x=\rho \times \sin\theta \times \cos\varphi \\ y=\rho \times \sin\theta \times \sin\varphi \\ z=\rho \times \cos\theta \end{array}\right.$$

**Figure 4:**
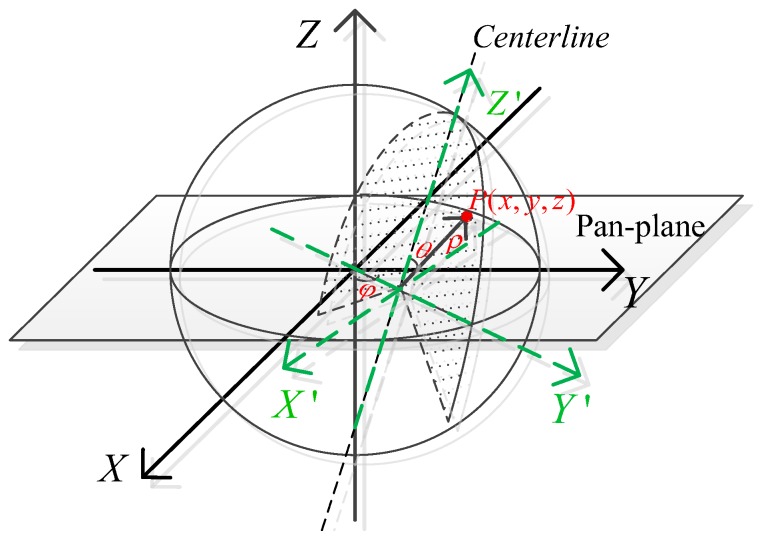
Coordinate system in the ideal situation.

Due to the installation error and structure of the product, the centers of the two devices would be in misalignment, and the centerline of the 2D LIDAR would bias away from upright, which results in the 3D scan being obtained in the rectangular coordinate system being skewed. Therefore, the coordinate system is defined as the Cartesian XYZ coordinate system whose origin is coincident with the center of the PTU, and the XY-plane indicates the Pan-plane of PTU as shown in Figure 4, whereas we define the LIDAR coordinate system of 2D LIDAR as the Cartesian $X^{\prime }Y^{\prime }Z^{\prime }$ coordinate system, which is separated from XYZ. The $Y^{\prime }Z^{\prime }$-plane means the scan plane of the 2D LIDAR.

**Figure 5:**
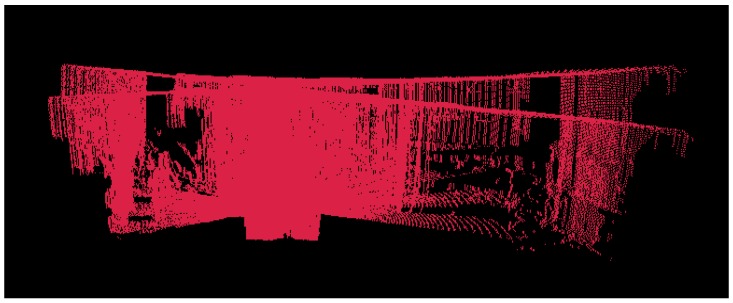
Original scan captured by our R2D-LIDAR system without calibration.

To solve this problem, the R2D-LIDAR system needs to be placed under the condition of the flat top of the roof or a horizontal plane over it. The bias of centerline would be solved first according to the length of the rays. Figure 5 is the point cloud data captured by our R2D-LIDAR system before calibration. The structure of the flat roof and ground produced a great deviation owing to the bias of the 2D LIDAR.

## 3. Calibration Model and Strategy

### 3.1. Measurement Model

**Figure 6:**
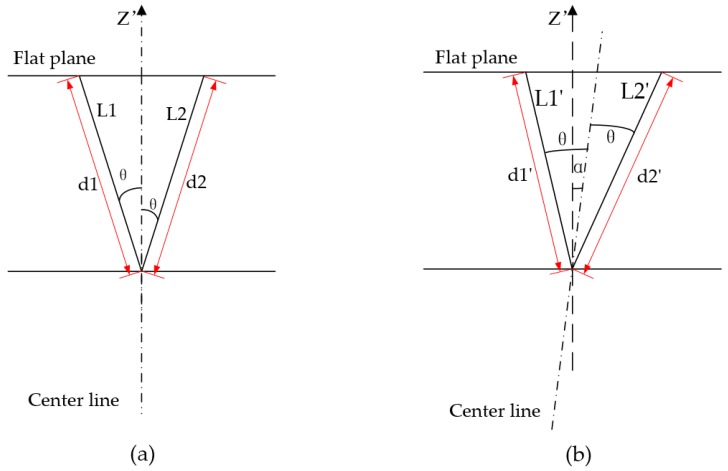
Geometrical relationship of calculating the bias angle. (**a**) denotes the centerline coincides with the $Z^{\prime }$ axis of $X^{\prime }Y^{\prime }Z^{\prime }$ coordinate system; (**b**) denotes the centerline deviates an angle α from $Z^{\prime }$ axis.

When only considering the $X^{\prime }Y^{\prime }Z^{\prime }$ coordinate system, if the centerline of the 2D LIDAR is perpendicular to the flat plane, the 2D scan will be divided uniformly by the centerline. Therefore, the ray length in one side should be equal to the corresponding ray length on the other side as presented in Figure 6a. L1 and L2 are the laser rays from the LIDAR center to the flat plane possessing equal angle with the centerline, and both lengths are equal to *d*. For the bias, the lengths of corresponding two rays are inequality as shown in Figure 6b. The centerline is shifted with angle *α*. $L1^{\prime }$ and $L2^{\prime }$ are the two laser rays symmetric with the centerline that deviated from the $Z^{\prime }$ axis in the $X^{\prime }Y^{\prime }Z^{\prime }$ coordinate system. In summary, the $X^{\prime }$, $Y^{\prime }$, $Z^{\prime }$ coordinates are indispensable to calibrating the bias. Since the PTU has Tilt rotation, the bias angle α could be expediently added to the Tilt to reduce the errors generated by the 2D LIDAR. However, as most of the rotating units just rotate around one direction, the bias angle could be adjusted online if the device is changed. It can be seen from Figure 6b that the angle of $L1^{\prime }$ turns to θ−α and the angle of $L2^{\prime }$ turns to θ+α. The length of $L1^{\prime }$ and $L2^{\prime }$ are $d1^{\prime }$ and $d2^{\prime }$, respectively. The bias angle α could be solved by the following equation:

(2) $$d1^{\prime }\times \cos\left(\theta -\alpha \right)=d2^{\prime }\times \cos\left(\theta +\alpha \right)$$

Since the length of the ray and the angle *θ* are easy to be received via the 2D LIDAR, the bias angle *α* can be calculated trivially. However, the inherent error of the LIDAR, the destabilization of the environment, and the bias angle *α* may not be the same for each line. Thereby, the results should be optimized through optimization methods such as the LM algorithm.

### 3.2. LM Optimized Algorithm

The LM algorithm was adopted to obtain the bias angle of the 2D LIDAR, and then found the transformation of the 2D LIDAR and PTU based on [15] after the bias adjustment.

The R2D-LIDAR system was placed in a flat plane environment and moved to different places to acquire the 3D point cloud data. The PTU rotated 360° to generate the two coincident scans at one time, which were defined as scan_front and scan_followed, respectively. The scan_front was used to calculate the bias angle through LM algorithm, while the scan_followed was employed to verify the bias calibration results.

The purpose of the adjustment of the 2D LIDAR is to correct the centerline accompanied by equating $L1^{\prime }$ and $L2^{\prime }$ as shown in Figure 6b. Assume that f(α) is the difference between $L1^{\prime }$ and $L2^{\prime }$ and can be expressed by:

(3) $$f\left(\alpha \right)=d1^{\prime }\times \cos\left(\theta -\alpha \right)-d2^{\prime }\times \cos\left(\theta +\alpha \right),$$

where $d1^{\prime }$ and $d2^{\prime }$ are the length of $L1^{\prime }$ and $L2^{\prime }$. The rays are chosen as an appropriate length by leaving out the rays whose angles *θ* are out of the appropriate range. This problem became an optimization problem to seek a bias angle

(4a) $$\alpha^{*}=\underset{\alpha }{\arg\min}\left\{F\left(\alpha \right)\right\},$$

where

(4b) $$F\left(\alpha \right)=\frac{1}{2}\sum_{i=1}^{m}\left(f{\left(\alpha_{i}\right)}^{2}\right).$$

The LM algorithm is a very efficient method to solve nonlinear least squares issues, and it is proposed by Levenberg and Marquardt to use a damped Gauss–Newton method [23]. Therefore, the LM algorithm is used to derive the minimum value. The summary of the calibration strategy of the bias angle is listed in Algorithm 1.

After the bias angle of the 2D LIDAR was modified, the method proposed by Hatem et al. [15] was applied to calibrate the deviation between the 2D LIDAR and PTU.

Algorithm 1: Bias angle calibration based on Levenberg-Marquardt method$\begin{array}{l} Input:\;\mathrm{Points}\;\mathrm{cloud}\;S\\ Output:\;\mathrm{Bias}\;\mathrm{angle}\;\alpha \;\mathrm{of}\;\mathrm{the}\;2D\;\mathrm{LIDAR}\\ \;1:\;Notations\\ \;2:\;\theta :\;\mathrm{the}\;\mathrm{angle}\;\mathrm{between}\;{\mathrm{LIDAR}}^{\prime }s\;\mathrm{ray}\;\mathrm{and}\;\mathrm{center}\;\mathrm{line}\\ \;3:\;D,D,\Theta :\;\mathrm{sets}\;\mathrm{of}\;d,\;d\;\mathrm{and}\;\theta \\ \;4:\;procedure\;\mathrm{FindBiasAngle}\left(S\right)\\ \;5:\;⊳Separate\;the\;coincident\;Point\;Cloud\;and\;extract\;the\;approriate\;rays\\ \;6:\;S_{1},S_{2}\leftarrow Separate(S)\\ \;7:\;D_{1}^{\prime },D_{2}^{\prime },\Theta \leftarrow ExtractRay(S_{1})\\ \;8:\;D_{3}^{\prime },D_{4}^{\prime },\Theta \leftarrow ExtractRay(S_{2})\\ \;9:\;⊳compute\;the\;Jacobian\;and\;Hessian\;of\;f(\alpha )\\ \;10:\;J\leftarrow Jacobian(f(\alpha ))\\ \;11:\;H\leftarrow Hessian(f(\alpha ))\\ \;12:\;⊳\;calculate\;the\;bias\;angle\;\theta \;through\;LM\;algorithm\\ \;13:\;\theta \leftarrow AngleCalculateLM(D_{1}^{\prime },D_{2}^{\prime },\Theta )\\ \;14:\;⊳verify\;the\;angle\;value\;and\;compute\;the\;line\;error\;in\;S_{2}\\ \;15:\;E_{2}\leftarrow LineErrorCalculate(\alpha ,D_{3}^{\prime },D_{4}^{\prime },\Theta )\\ \;16:\;if\;E_{2}>\varepsilon \\ \;17:\;compute\;\alpha \;again\\ \;18:\;else\\ \;19:\;\mathrm{return}\;\alpha \\ \;20:\;end\;procedure \end{array}$

## 4. Experiments

The R2D-LIDAR system was applied to obtain 3D scans. A scan contains 37,000 points of the surroundings. The values of *θ* within a proper range were adopted to calculate the bias angle *α*. Firstly, we employed the simulation to verify the theoretical completeness of the improved calibration method. After that, several 3D scans were captured in three typical scenarios to prove the efficiency of adding bias adjustment. Then, the comparison between our method and Alismail’s method [15] was carried out, while our method is improved based on the Alismail’s method. At last, the accuracy of the point clouds after calibration was presented.

### 4.1. Simulation

In this part, the goal is to validate the theoretical feasibility and veracity of the improved calibration method in controlled circumstances. A synthetic bias of the 2D LIDAR was generated by the Tilt rotating of the PTU. After differing the total bias angles with and without the synthetic bias through

(5) $$\alpha_{adding}=\alpha_{total}-\alpha .$$

The adding bias angle could be estimated to compare with the setting value. A stable and similar value could prove the validity of our calibration method.

**Figure 7:**
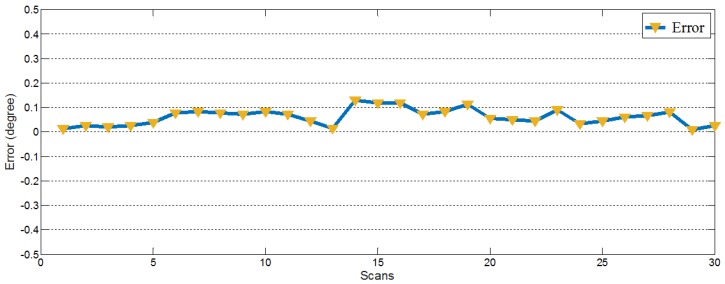
Absolute errors between the calculated adding bias angle and the setting angle. The error varies in a small range.

In the experiment, we set the adding bias angle to be 5 degrees. After that, several scans with synthetic bias were collected to calculate the value of the bias angle. As illustrated in Figure 7, the absolute errors between the calculated value and the setting value were within the range from 0.0095° to 0.1299°. Therefore, the proposed calibration method is accurate and stable to obtain the deviation of the 2D LIDAR. Whereafter, a few demonstrations using real data would be illustrated in the following experiments.

### 4.2. Calibration in Different Scenarios

**Figure 8:**
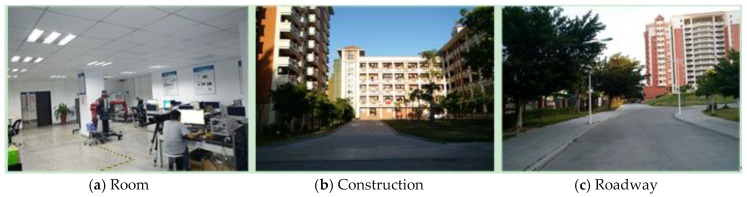
Three typical scenarios (room, construction, and roadway) were adopted to capture the 3D point clouds.

Our improved calibration method could obtain the bias angle of the 2D LIDAR directly. Therefore, it could not only adjust the point clouds offline but also be employed in the capture program in advance. In this experiment, our R2D-LIDAR system with and without bias adjustment was adopted to capture the point clouds in some typical scenarios with an obvious geometry such as room, construction, and roadway as shown in Figure 8. All the point clouds were collected in the same set-up of the R2D-LIDAR system. The bias angle of value 3.75° was added when using a calibrated capture program.

**Figure 9:**
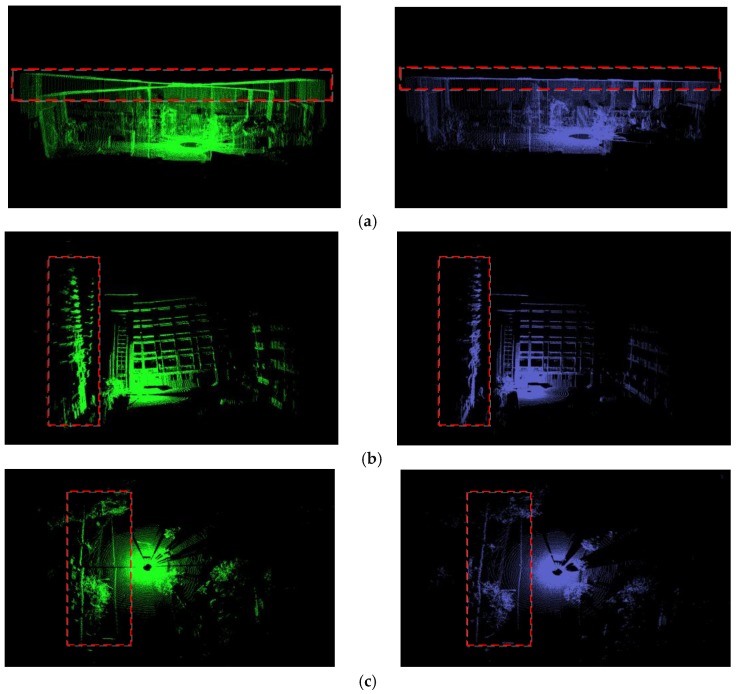
3D point clouds captured by the R2D-LIDAR system in three typical scenarios, i.e., room, construction and roadway. The left plots present the uncalibrated point clouds, and the right plots represent the point clouds captured with bias adjustment. The differences between the left plots and the right plots highlighted by red box: (**a**) the great bias of the roof on the left was eliminated after calibrating and some bumps on the right were the lamp holders on the roof; (**b**) the construction on the left was obviously an inclination, which was rectified through our adjustment; (**c**) the roadway existed crack on the left and it became continuous after adjustment.

Figure 9 presents the point clouds of the three scenario from different perspectives. As indicated by the red box, the deviations have been predominantly adjusted both vertical and horizontal directions. Accordingly, the coordinates of the point clouds have been corrected especially for the later registration process. Hence, it is very effective and convenient to apply the improved calibration result in the 3D point clouds capture system. As shown in Figure 9, the calibration parameters succeed in common scenarios.

### 4.3. Comparison with Alismail’s Work

To prove the superiority of our calibration method, our improved calibration strategy was also compared with Alismail’s method [15]. Alismail’s work fully considered the spinning between the 2D LIDAR and the rotating unit. A full scan captured through 360° rotating of the rotating unit was used to estimate the calibrating transform. Therefore, the full scan could be split into two scans possessing the same sampled surfaces. According to minimization of the cost function of the difference between these two point sets, the calibrating transform was calculated for the subsequent point cloud calibration.

**Figure 10:**
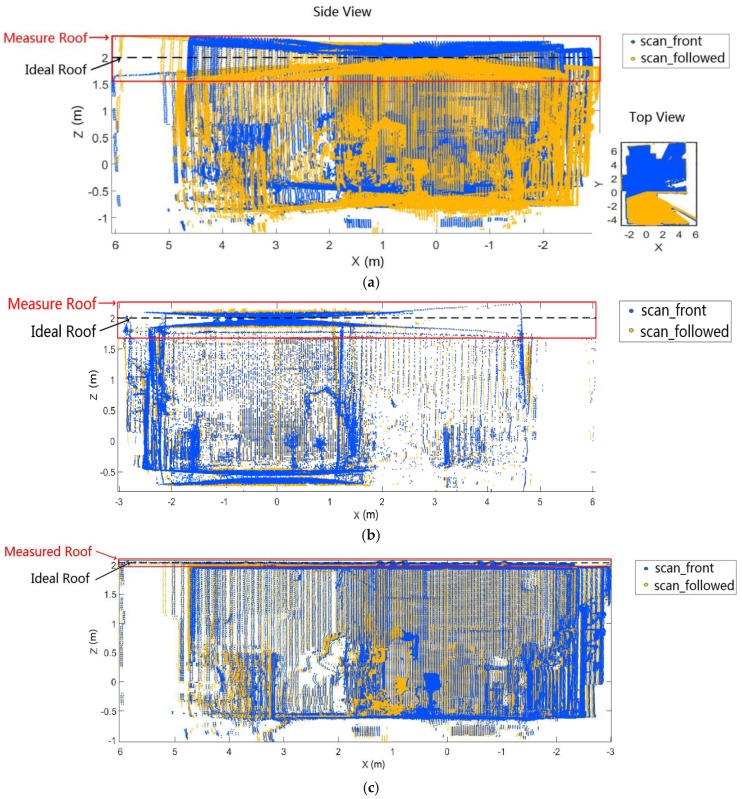
(**a**) is the full scan captured by our R2D-LIDAR system with a large deviation. The full scan contains two coincident scans of the point clouds marked by blue and yellow; (**b**) is the calibration result using Alismail’s method. Alismail’s method is effective in the two coincident scans matching but fails in bias adjustment, which is reflected in the roof; (**c**) is the calibration result employing both bias adjustment of the 2D LIDAR and Alismail’s method. The performance demonstrates the superiority of our improved calibrating method compared with Alismail’s method.

However, it is hard to adjust the point cloud by simply adding the calibrating transform if the 2D LIDAR has generated the bias in the scanning direction. Figure 10 depicts the scan and the corresponding calibration results with and without bias adjustment. Figure 10a presents the full scan collected by the R2D-LIDAR system with a 360° rotating angle. Visibly, a large deviation exists in the full scan. As for the captured principle of the R2D-LIDAR system, the full scan contains two coincident scans of the surroundings as illustrated in two colors. Figure 10b is the calibration result calculated through Matlab (2016b, MathWorks, Natick, MA, USA) and it only adopted Alismail’s method. We can see that some deviations were still unvaried and it failed to adjust the scan. Figure 10c displayed the calibration results, which applied both bias adjustment of the 2D LIDAR and Alismail’s method. The roof was flattened after adding the bias adjustment while some humps were caused by the overhead light.

Moreover, the improvement using our calibration strategy is indicated according to the roof deviation between the measured roof and the ideal roof. The ideal roof is 2 m in the $X^{\prime }Y^{\prime }Z^{\prime }$ coordinate system as shown in Figure 10. The roof deviations calculated by the mean of the distances in Figure 10a–c are 0.0934 m, 0.0624 m and 0.0188 m, respectively. The roof deviation adjustment only adopted Alismail’s method improved 33.2% while that adopting both our calibration method and Alismail’s method improved 80%. The scan was successfully adjusted with our calibrating parameters, which proved the superiority of the improved calibrating method compared with Alismail’s method.

### 4.4. Accuracy of Calibration Result

**Figure 11:**
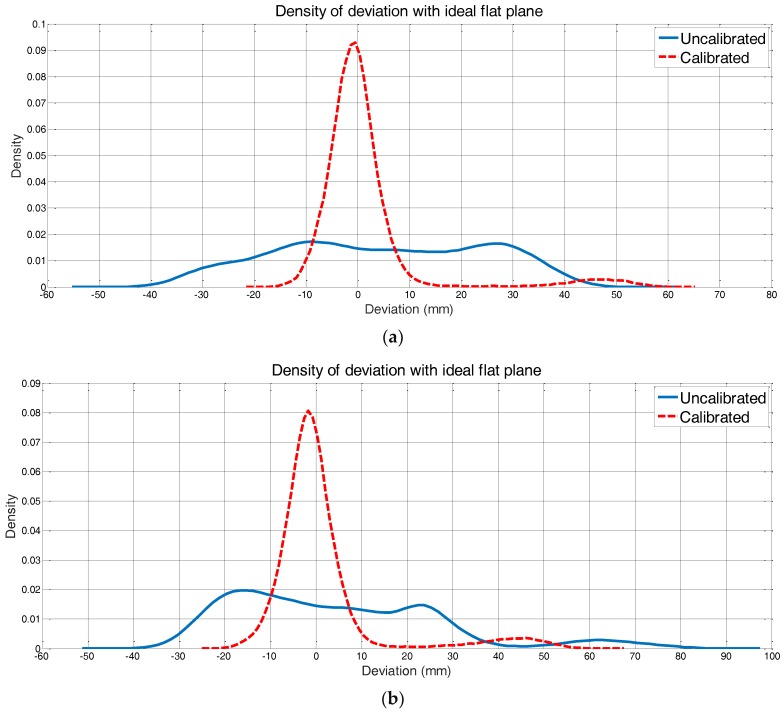
(**a**,**b**) are the deviation comparisons of scan_front and scan_followed, respectively. The bias angle was estimated from scan_front. The deviation under calibration condition in (**a**) is mainly ranging from −15 mm to 15 mm and the deviation under calibration condition in (**b**) is mainly ranging from −20 mm to 15 mm. Both deviations after calibration were greatly decreased compared to the uncalibrated condition.

For persuasive assessment, the deviation of the ideal plane was calculated to assess the accuracy of our method. Figure 11a,b demonstrated the reduction of deviation after 2D LIDAR calibration through the histograms. The histogram was estimated by kernel density estimation inspired by [19]. As previously mentioned, the bias angle was computed according to scan_front and put back to the same scan afterward. The densities of the deviation assigning to scan_front calibrated and uncalibrated were exhibited in Figure 11a. We could find that the deviation is mainly ranging from −15 mm to 15 mm, which was greatly decreased after calibration. Figure 11b was the deviation comparison of scan_followed. The variation range was a little broader than scan_front but was still an accurate consequence.

The quantitative evaluation demonstrated the validity and necessity of the proposed calibration strategy.

## 5. Conclusions

In this work, a novel approach to calibrate sensor offsets before transformation between capturing drivers was proposed. Using a flat plane allows us to estimate the sensor offset primitively within the strict theoretical framework of the LM algorithm. For the case of R2D-LIDAR system calibration, it was beneficial for adjusting sensor offset first since the bias angle that affects the transformation from spherical coordinates to rectangular coordinates behaves in a nonlinear fashion. The adjustment reduced the complexity of computing the transformation between different devices. Furthermore, the bias angle can be conveniently added to the data collecting program.

As for future work, more comparative experiments should be conducted to validate the robustness of this method. In addition, we will apply the calibration results in the registration of scans, and we strongly believe that the performance will be enhanced significantly.
